# The bloom and the breathless: the rising burden of pollen allergy

**DOI:** 10.1016/j.eclinm.2025.103327

**Published:** 2025-06-23

**Authors:** eClinicalMedicine

As we mark World Environment Day on June 5, understanding how climate change impacts human health is ever more urgent and essential to protect vulnerable populations. One effect gaining attention from both the public and scientific community is the link between changing climate, increased pollen production, and the rising burden of allergic diseases. Pollen is an allergen that can cause allergic rhinitis, with or without allergic conjunctivitis, and exacerbate asthma.

Allergic rhinitis is characterised by type 2 inflammation of the lining of the nose manifesting as rhinorrhoea, sneezing, nasal blockage and itching of the nose. Allergic rhinitis has been considered for long time a localised disorder, but evidence suggests that it can involve the entire respiratory tract. This theory is also supported by the frequent coexistence of rhinitis and asthma. Estimates indicate that allergic rhinitis affects approximately 10–30% of adults worldwide, with higher prevalence observed in urban areas due to factors like pollution and lifestyle changes. Determining the global prevalence of asthma remains challenging due to variations in diagnostic criteria and reporting practices. Current estimates suggest that approximately 260 million people worldwide are affected by asthma. The majority of cases begin in childhood, probably driven by underlying genetic predispositions, but environmental factors also play a role, with early-life exposure to pollen identified as a substantial risk factor for the development of the condition in childhood.

Allergic rhinitis and asthma can strongly impair quality of life during the pollen season, although to varying degrees of severity as they can disrupt sleep, concentration, and overall comfort. Asthma attacks can lead to wheezing, breathlessness, chest tightness, and severe coughing. These respiratory symptoms can become more frequent and acute during pollen season and limit physical activity and might induce a sense of fear of sudden attacks. The necessity to avoid certain environments or restrict outdoor activity can lead to social isolation, particularly in children and adolescents who would benefit from sports or recreational activities during the warmer months.

Rising temperatures and increased atmospheric carbon dioxide concentrations stimulate plant growth and augment pollen production, extending pollen seasons and increasing its concentrations. Changing climate patterns also affect the geographic distribution and seasonality of pollen and other airborne allergens, and, as a result, people with allergies are facing longer periods of exacerbated symptoms.

Additionally, the increased frequency and severity of extreme weather events, such as thunderstorms and heavy downpours, have an important role in pollen related conditions. Thunderstorms can fragment pollen grains, making them more allergenic, as smaller particles can penetrate deeper into the airways, reaching the lower respiratory tract. Thunderstorm asthma refers to the sudden onset of acute asthma attacks following a thunderstorm during pollen season. Such outbreaks have been reported globally and can rapidly overwhelm health-care systems, posing serious risks to individuals with pollen allergies.

Medications can alleviate immediate symptoms and, in some cases, address the underlying allergic inflammation to prevent long-term complications. For symptomatic relief, oral and intranasal antihistamines are commonly used due to their effectiveness and relatively manageable side effects, like drowsiness. Intranasal corticosteroids are considered the most effective first-line treatment for moderate to severe rhinitis but when symptoms remain uncontrolled, combination sprays containing both a corticosteroid and an antihistamine should provide a superior alternative. For patients with both asthma and allergic rhinitis, leukotriene-receptor antagonists offer dual benefit by controlling both nasal and bronchial symptoms and reducing inflammation and bronchoconstriction. For long-term management, allergen specific immunotherapy is the only disease modifying treatment.

Climate-driven changes in airborne allergens and associated allergic reactions are projected to increase up to 200% by 2050 in Europe, which is already experiencing pollen bombs more frequently, and could affect allergic rhinitis and asthma prevalence. Urbanisation and associated increased levels of air pollution and the reductions in biodiversity are strongly influencing pollen concentrations and increasing its allergenicity due to repeated exposure to the same allergenic plants, particularly those that dominate urban landscapes. Although tackling global warming and promoting biodiversity would be the ideal strategy, urban planning could also contribute to mitigation measures. It is impossible to reduce pollen exposure to levels that are harmless for individuals with allergies, and setting a safety threshold remains particularly challenging due to the high variability in individual sensitivity, but the establishment of green spaces with hypoallergenic tree species and control of allergenic invasive weeds could reduce pollen exposure. Pollen forecasts and information services via the use of technology and mobile apps can also provide effective tools for allergen avoidance and management of symptoms.

Despite therapeutic and technological advances, pollen allergy remains underdiagnosed and frequently undertreated. Raising awareness, among both the public and health-care professionals will be crucial to ensuring timely diagnosis and appropriate management. Recognising pollen allergy as a growing public health issue will be essential to protect individuals with allergies and reduce the related burden of respiratory diseases. A multidisciplinary approach that integrates clinical care and pharmacological advance, urban design and environmental policies together with public education and awareness, will be key to building healthier communities and improve quality of life for all.

